# Visual Monitoring of Fatty Acid Degradation during Green Tea Storage by Hyperspectral Imaging

**DOI:** 10.3390/foods12020282

**Published:** 2023-01-07

**Authors:** Yiyi Zhang, Lunfang Huang, Guojian Deng, Yujie Wang

**Affiliations:** State Key Laboratory of Tea Plant Biology and Utilization, Anhui Agricultural University, Hefei 230036, China

**Keywords:** green tea storage, chemical imaging, fatty acids, prediction models, visualization map

## Abstract

The reduction in freshness during green tea storage leads to a reduction in its commercial value and consumer acceptance, which is thought to be related to the oxidation of fatty acids. Here, we developed a novel and rapid method for the assessment of green tea freshness during storage. Hyperspectral images of green tea during storage were acquired, and fatty acid profiles were detected by GC–MS. Partial least squares (PLS) analysis was used to model the association of spectral data with fatty acid content. In addition, competitive adaptive reweighted sampling (CARS) was employed to select the characteristic wavelengths and thus simplify the model. The results show that the constructed CARS-PLS can achieve accurate prediction of saturated and unsaturated fatty acid content, with residual prediction deviation (RPD) values over 2. Ultimately, chemical imaging was used to visualize the distribution of fatty acids during storage, thus providing a fast and nondestructive method for green tea freshness evaluation.

## 1. Introduction

Fatty acids are an important group of substances in agri-food products and are divided into saturated fatty acids (SFAs) and unsaturated fatty acids (UFAs) [1]. As important nutritional components of agri-food products, fatty acids are chemically unstable and usually undergo various chemical changes during storage, including hydrolysis, rancidity, and auto-oxidation [2,3]. For example, fatty acids are oxidized into various carbonyl compounds, mainly aldehydes and ketones [2]. Oxidative degradation of fatty acids is also a major cause of reduced freshness during the storage of agricultural products [3]. The commercial value of green tea, as a high value-added agricultural product, is usually determined by its freshness. Usually, the fresher green tea is, the higher the commercial value, while green tea that has lost its freshness is considered cheap and undesirable. During storage, the fatty acids responsible for the aroma of the tea break down into aldehydes and ketones. The production of these aromatic substances leads to a reduction in the aroma quality of green tea, thus affecting commercial value and consumer acceptance. Therefore, it is necessary to establish a method for the determination of green tea freshness with fatty acids as target substances.

The current gold standard for fatty acid determination in tea is gas chromatography–mass spectrometry (GC–MS) [4,5]. A green tea sample undergoing pretreatment, such as grinding and digestion, is analyzed using professional precision instruments, and finally, an accurate fatty acid profile is obtained. However, this method is time-consuming and costly and requires the consumption of large amounts of volatile solvents, which can easily cause environmental pollution and pose a health risk to operators. Therefore, GC–MS detection cannot meet the demand for rapid and green detection of fatty acids in green tea in storage. Near-infrared (NIR) spectroscopy is a nondestructive and green analytical technique capable of detecting substances containing hydrogen groups, including fatty acids [6,7]. However, NIR spectroscopy can detect only one point in the sample and cannot obtain spatial information on the target substance. As an alternative, chemical imaging, also known as hyperspectral imaging (HSI) [8], has received attention from researchers due to its advantages of being fast and nondestructive and chemical-free, and is expected to enable the detection of fatty acids in agricultural products [9]. Chemical imaging combines the functions of spectral detection and image analysis, enabling rapid detection of components in complex agricultural product matrices. More importantly, chemical imaging visualizes the distribution of target components, such as fatty acids, by means of image analysis [9]. For example, changes in saturated and unsaturated fatty acids during pork processing have been determined using chemical imaging [10]. However, the potential of chemical imaging based on optical techniques for the detection of fatty acid profiles in tea samples has not been explored.

Therefore, the aim of this study was to investigate the potential of chemical imaging for the detection of fatty acid profiles in green tea during storage and ultimately to establish a rapid visual monitoring method for storage freshness of green tea. Specifically, this study intended to (1) prepare green tea samples with different storage times, (2) detect fatty acid profiles in different green tea samples by GC–MS, (3) develop and validate prediction models for fatty acids, and (4) map the distribution of fatty acids in green tea during storage using chemical imaging.

## 2. Materials and Methods

### 2.1. Preparation and Collection of Tea Samples

Newly produced Huangshan Maofeng green teas were purchased from the Xieyuda Tea Company located in Huangshan City in April 2020. Upon receipt, the green tea samples were examined carefully by professional tea panelists to ensure that they were completely fresh. Subsequently, all the green teas were stored in a stable environment at a room temperature of 25 °C. When the storage time reached 30 days, 60 days, 90 days, 120 days and 150 days, a portion of the green tea samples was removed. After undergoing sensory evaluation, they were placed in sealed bags and subsequently placed in a refrigerator at a low temperature of −80 °C. Since the results of the sensory evaluation showed significant aging and lack of freshness when the green tea was stored for 150 days, the storage was cut off at 150 days. For each sampling period, 15 samples were randomly removed from the stored samples so that a total of 90 green tea samples were collected for different storage periods.

### 2.2. Sensory Evaluation of Tea Samples

All green tea samples were taken from the refrigerator and then evaluated by a sensory panel consisting of five panelists according to the method described in GB/T 23776-2018 (methodology for sensory evaluation of tea). The freshness of the green teas was divided into fresh, slightly fresh, and not fresh. The results of the evaluation show that green teas were fresh when stored for no more than 60 days (from 0 days to 30 days). When the storage time ranged from 60 days to 120 days, the green teas were slightly fresh. A storage time of 150 days indicated a state of not being fresh.

### 2.3. Acquisition and Processing of Hyperspectral Images

#### 2.3.1. Hyperspectral Imaging Instrument

After sensory evaluation, all green tea samples were used for the acquisition of hyperspectral images. Here, a hyperspectral imaging instrument was employed [11], which consists of one spectrometer (ImSpector N17E, Spectral Imaging Ltd., Oulu, Finland) in the wavelength range of 900–1700 nm, one CCD camera (Imperx Inc., Boca Raton, FL, USA), one camera lens (Schneider Kreuznach, Bad Kreuznach, Germany), and two arrays of light sources (Illumination Technologies Inc., New York, NY, USA). The collection of hyperspectral images was performed in a line-scanning configuration. During collection, all parameters of the instrument were set as described in Wang et al. [11].

#### 2.3.2. Image Correction and Data Extraction

The acquired raw images contain noise introduced by mechanical vibration of the instrument and interference of light-intensity changes. Therefore, standard references were used to correct the raw images. Then, spectral image (Isuzu Optics Corp., Taiwan, China) software was employed to correct the raw hyperspectral images by using a black and a white reference according to Wang et al. [11]. The corrected images were loaded into Python software. In this software, the region where the tea sample is located is used as the region of interest (ROI), while the background information is eliminated. Due to the difference in spectral absorption values in the tea sample and the background, a simple masking method was used to remove the background by setting a threshold value of 0.1.

### 2.4. Determination of Fatty Acid Profiles

The fatty acid content was determined by GC–MS with reference to the method in the literature [12,13]. Frozen tea powder (0.1 g) was accurately weighed and added to the extraction solution (prepared from n-hexane and isopropanol in a ratio of 3:2 by volume) and Na_2_SO_4_ solution. The above solution was vortexed, followed by centrifugation at 4× *g* °C and 12,000 rpm for 15 min, and the supernatant was collected. The lower residue was added to 0.5 mL of extraction solution and vortexed again, and the supernatants were combined. The collected supernatant was blown to dryness with nitrogen, and after complete drying, 3 mL of methylation reagent (methylation reagent was made from methanol, toluene and concentrated sulfuric acid in a ratio of 88:10:2 by volume) was added and then removed from the water bath at 80 °C for 1 h. After cooling to room temperature, 1 mL of heptane was added and vortexed, and the extracted upper solution was transferred to a 2 mL centrifuge tube. The extraction was repeated, and the volume was fixed with heptane to 2 mL. A 5 mL centrifuge tube was used, 0.4 g of anhydrous sodium sulfate was added, and the solution was poured into the volume and shaken well to remove the residual water. Fatty acids were quantified by adding internal standards as reference values, including palmitic acid, stearic acid, and arachidic acid, and five unsaturated fatty acids, 9-hexadecenoic acid, oleic acid, linoleic acid, linolenic acid, and cis-11-eicosenoic acid.

### 2.5. Establishment and Validation of Quantitative Models

Principal component analysis (PCA) was employed to observe clustering trends at different storage times by using fatty acid profiles and spectral data, respectively. PCA is a widely used statistical method for data reduction and data visualization.

In this study, the correlation between spectral information and target fatty acid content was achieved by building a partial least squares (PLS) model. PLS is one of the most widely used linear algorithms for solving regression problems and has advantages in dealing with high-dimensional spectral data [14,15,16]. The PLS algorithm considers both the spectral matrix and the target matrix. In PLS, the spectral data are projected into new variables called latent variables (LVs) that provide the maximum covariance between the spectral matrix and the target matrix. In this study, the number of LVs was optimized by 5-fold cross-validation. The regression coefficients (RCs) of the PLS model indicate the contribution of the wavelength involved in the calibration [10]. A wavelength possessing higher RCs in absolute value is usually considered to be contributing. In addition, characteristic wavelengths were selected using CARS [17], and the simplified CARS-PLS model was then established.

All samples were first divided into calibration and prediction sets in a 2:1 ratio. Sixty samples from the calibration set were used to build the prediction model, and the remaining 30 samples from the prediction set were used to validate the prediction accuracy of the model. The performance of the PLS model was evaluated using the correlation coefficients of calibration (Rc) and prediction (Rp), the root means square error of calibration (RMSEC) and prediction (RMSEP), and the residual prediction deviation (RPD). The RPD is obtained by calculating the ratio of the standard deviation (SD) and RMSEP of the prediction set samples. All modeling operations were achieved using MATLAB (R2014a, Mathworks Inc., Natick, MA, USA).

### 2.6. Visualization of Fatty Acid Profiles

In addition to quantitative prediction of the components, chemical imaging can also show the distribution of the components in a sample, which is better than the traditional spectroscopic techniques [10]. With the optimal prediction model developed, chemical imaging introduces the spectral fingerprint of each pixel in the hyperspectral image into the prediction model. Then, the content of the target component at each pixel is obtained. Finally, the distribution of the components is mapped as a pseudo-color image. These operations were implemented using MATLAB (R2014a, MathWorks Inc., Natick, MA, USA).

## 3. Results and Discussion

### 3.1. Fatty acid Reduction during the Storage of Green Tea

**Figure 1:**
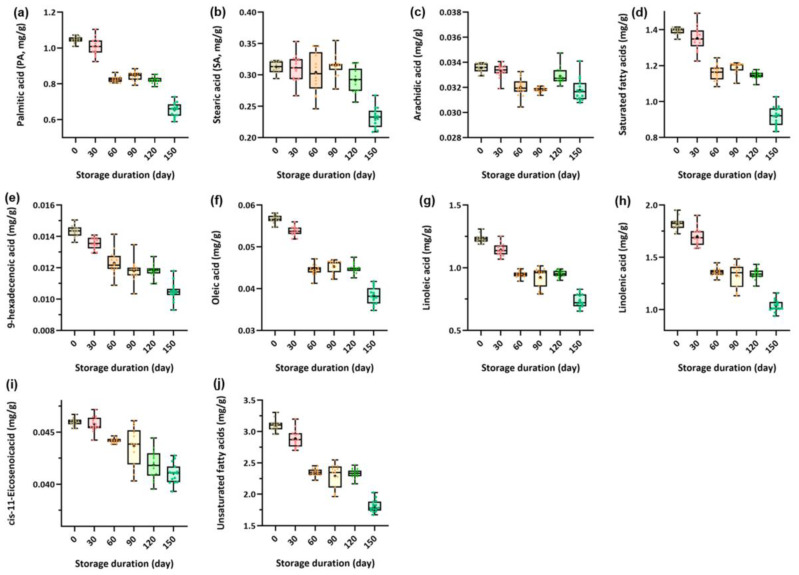
Measured fatty acids of tea samples with different storage duration. The boxes are bounded by the 25% and 75% quartiles, with the median values inside, whereas the extreme levels correspond to 5% and 95% quartiles. (**a**) palmitic acid; (**b**) stearic acid; (**c**) arachidic acid; (**d**) total saturated fatty acids; (**e**) 9-hexadecenoic acid; (**f**) oleic acid; (**g**) linoleic acid; (**h**) linolenic acid; (**i**) cis-11-eicosenoic acid; and (**j**) total unsaturated fatty acids. Different colors represent tea samples with different storage duration.

The fatty acid profiles in green tea samples from different storage durations were detected using the GC–MS method, and the results are shown in Figure 1. Specifically, three saturated fatty acids, including palmitic acid, stearic acid, and arachidic acid, and five unsaturated fatty acids, including 9-hexadecenoic acid, oleic acid, linoleic acid, linolenic acid, and cis-11-eicosenoic acid, were detected and quantified. From Figure 1, the content of most of the nine fatty acids decreases sharply during storage, especially palmitic acid (Figure 1a), 9-hexadecenoic acid (Figure 1e), oleic acid (Figure 1f), linoleic acid (Figure 1g), linolenic acid (Figure 1h), and cis-11-eicosenoic acid (Figure 1i). The trends of total saturated (Figure 1d) and unsaturated fatty (Figure 1j) acids during storage were consistent. There was a slight decrease in content from 0–30 days, while a sharp decrease was observed at 60 days and remained slightly lower for the next 60 days before another sharp decrease was observed at 150 days. These results are consistent with the results of sensory evaluation, where green tea was fresh after 0–30 days of storage, slightly fresh from 60–120 days, and not fresh when storage reached 150 days.

**Figure 2:**
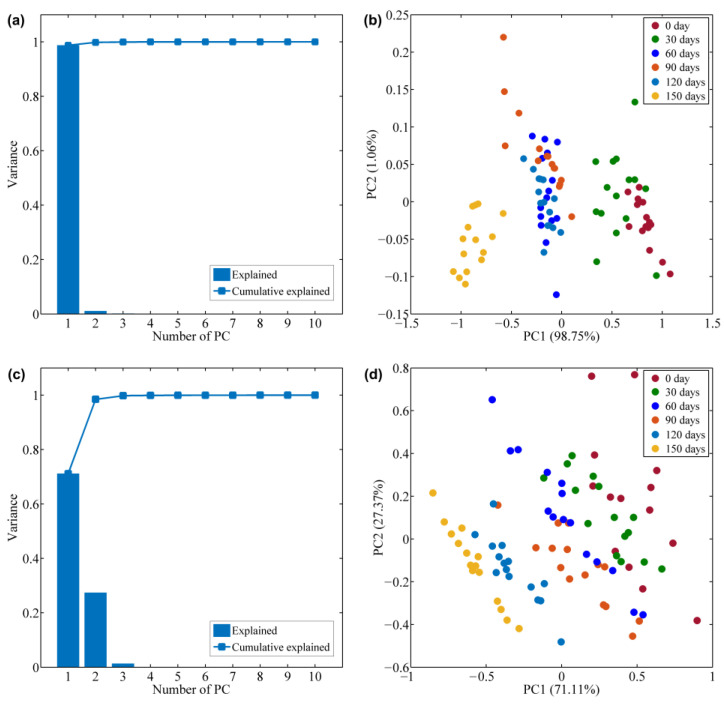
Explained and cumulative explained variance by PCA based on measured fatty acids (**a**) and spectral data (**c**). PCA score plots of tea samples with different storage duration based on measured fatty acids (**b**) and spectral data (**d**).

Furthermore, fatty acid profiles were used for principal component analysis (PCA) to observe clustering trends at different storage times. Figure 2a,b give the explained and cumulative explained variance by PCA based on measured fatty acids by GC-MS, respectively, while Figure 2c,d give the explained and cumulative explained variance by PCA based on measured spectral data by HSI, respectively. Figure 2a,b show the variance explained by different principal components and the score plots of all green tea samples under the first two principal components (PC1 and PC2), respectively. PC1 and PC2 explained 98.75% and 1.06% of the total variance, respectively; thus, over 99% of the variance could be explained by the first two PCs. The score plots indicate that the green tea samples with storage durations of 0 days and 30 days, storage durations of 60, 90, and 120 days, and storage durations of 150 days were completely separate. Thus, the score plot of the fatty acid profile implies the variation in green tea freshness.

### 3.2. Spectral Analysis during the Storage of Green Tea

Figure S1 shows the spectral curves in the wavelength range of 900–1700 nm of all green tea samples. Each spectral curve was obtained from a tea sample, which was obtained by calculating the average spectral values of all pixels within the ROI. All samples showed a similar shape and differed only in the absorbance intensity along the spectral region. Specifically, distinct absorptions were shown in the range of 1160–1180, 1450–1500 and 1650–1660 nm in the spectral profile. The absorption of 1160–1180 nm may be due to the overtone caused by amino acids. While the absorption of 1450–1500 nm is associated with the O-H first overtone, which is related to water [18]. And the absorption of 1650–1660 nm corresponds to the overtone caused by caffeine [19]. These substances are the main components in tea. In contrast, the absorption peaks of fatty acids in tea have not been reported. It is known from other agricultural products that the absorption peaks of fatty acids may be in the range of 1100–1400 nm [20,21]. These absorption peaks were not observed in this study due to the low content of fatty acids in tea samples.

Figure 2a,b give the explained and cumulative explained variance by PCA based on measured fatty acids by GC-MS, respectively, while Figure 2c,d give the explained and cumulative explained variance by PCA based on measured spectral data by HSI, respectively. The PC1 and PC2 explained 71.11% and 27.37% of the total variance, respectively; thus, over 98% of the variance could be explained by the first two PCs. From Figure 2d, the green tea samples showed regular clustering at different storage times, implying that the spectral information can indicate the freshness of green tea. In addition, the results of the fatty acid-based PCA score plot were consistent with those of the score plot based on spectral data, implying that changes in fatty acids during the storage of green tea can be described by spectra.

### 3.3. Prediction of Fatty Acids Using All Spectral Signals

**Figure 3:**
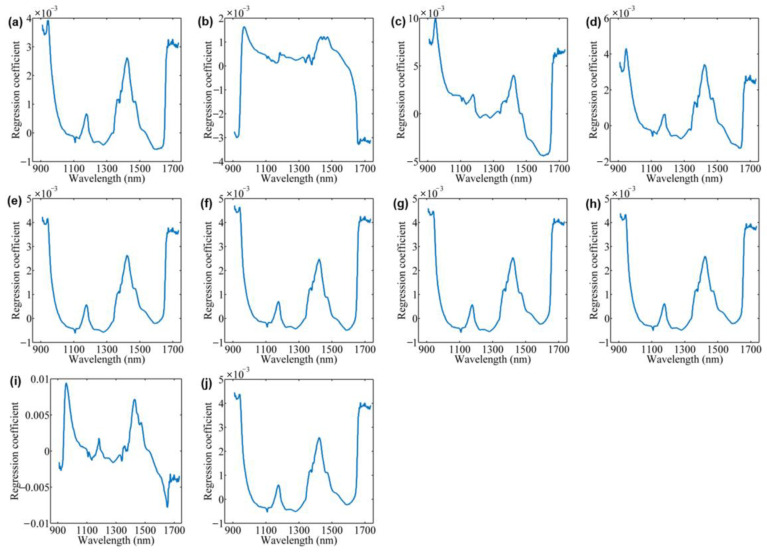
Regression coefficient plots of PLS models for the prediction of fatty acids: palmitic acid (**a**); stearic acid (**b**); arachidic acid (**c**); total saturated fatty acids (**d**); 9-hexadecenoic acid (**e**); oleic acid (**f**); linoleic acid (**g**); linolenic acid (**h**); cis-11-eicosenoic acid (**i**); and total unsaturated fatty acids (**j**).

The PLS models for fatty acid prediction using spectral data in the wavelength range of 900–1700 nm are given in Table 1. For calibration, the PLS models achieved acceptable performance with Rc values of 0.8561, 0.8120, 0.8310, 0.8732, 0.8829, 0.8624, 0.8643, 0.8189, and 0.8653 for palmitic acid, arachidic acid, total saturated fatty acids, 9-hexadecenoic acid, oleic acid, linoleic acid, linolenic acid, cis-11-eicosenoic acid, and total unsaturated fatty acids, respectively. When the samples in the prediction set were used for model validation, the PLS models yielded lower Rp values, with RPD values of 1.58, 0.98, 1.49, 1.49, 1.91, 1.84, 1.96, 1.60, and 1.93. For quantitative models, the RPD value is a key evaluation metric. RPD values over 2.0 indicate accurate performance of the developed model [22]. Thus, all the established PLS models were unable to achieve accurate predictions for various fatty acids, as the RPD values of all models were less than 2.0. Such results are to be expected. The full spectrum is composed of the absorbance/reflectance at 508 wavelengths. The correlation of the values at different wavelengths with the target components is differential, i.e., some of these wavelengths contribute a lot to the component prediction, while the remaining wavelengths contribute a little. Therefore, the RC values of the PLS model are plotted to observe the contribution of each wavelength, as shown in Figure 3. For most fatty acids, the RC curves showed a similar shape and differed only in the values along the spectral region, such as palmitic acid, arachidic acid, total saturated fatty acids, 9-hexadecenoic acid, oleic acid, linoleic acid, linolenic acid, cis-11-eicosenoic acid, and total unsaturated fatty acids. High RC values were observed in the spectral ranges of 930–960 nm, 1140–1160 nm, 1410–1430 nm, and 1650–1680 nm. This may be because the various fatty acid monomers have similar chemical structures and functional groups. However, whether these bands are associated with fatty acids in tea leaves still needs to be further explored, although in other studies the absorption peaks in the range of 1100–1400 nm were considered to be associated with fatty acids [20,21].

### 3.4. Prediction of Fatty Acids Using Characteristic Spectral Signals

**Figure 4:**
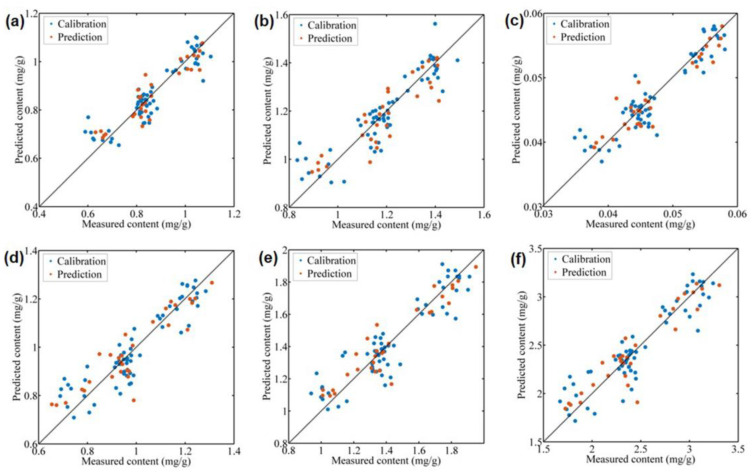
Optimal prediction models using CARS-PLS on spectral data for palmitic acid (**a**); total saturated fatty acids (**b**); oleic acid (**c**); linoleic acid (**d**); linolenic acid (**e**); and total unsaturated fatty acids (**f**).

The CARS-PLS models were then established on the selected wavelengths, and the results are shown in Table 1. For saturated fatty acids, the CARS-PLS models gave satisfactory performance for the prediction of palmitic acid and total saturated fatty acids, with Rc values of 0.9112 and 0.9011, Rp values of 0.9283 and 0.8961, and RPD values of 2.73 and 2.26. The CARS-PLS models failed to predict the content of steric acid and arachidic acid, with low RPD values of 1.17 and 1.13, respectively. For unsaturated fatty acids, the CARS-PLS models achieved satisfactory performance for most kinds of fatty acids, except for 9-hexadecenoic acid and cis-11-eicosenoic acid. The RPD values for oleic acid, linoleic acid, linolenic acid, and total unsaturated fatty acids reached 3.14, 2.43, 3.01, and 2.78, respectively. Overall, the CARS-PLS models achieved accurate prediction of six fatty acid indicators based on the selected characteristic spectral variables. The comparison between the predicted results and the measured results is shown in Figure 4. It can be observed from Figure 4 that all samples have similar measured and predicted values, indicating high prediction accuracy.

Spectral selection is an important step in NIR modeling to simplify the modeling process and improve the accuracy of the model. CARS, a commonly used variable selection method, has been shown to be advantageous in enhancing model accuracy. In this study, CARS was used to select the characteristic wavelengths associated with fatty acids from 508 wavelengths, and the number was reduced to 6–48, which means that at least 90.55% of irrelevant variables were eliminated. From Table 1, when the PLS models were compared with the CARS-PLS models, the accuracies were improved to different degrees for all indicators, especially for palmitic acid (RPD values of 1.58 vs. 2.73), total saturated fatty acids (1.49 vs. 2.26), oleic acid (1.91 vs. 3.14), linoleic acid (1.84 vs. 2.43), linolenic acid (1.96 vs. 3.01), and total unsaturated fatty acids (1.93 vs. 2.78).

### 3.5. Visualization of Fatty Acid Degradation during Green Tea Storage

**Figure 5:**
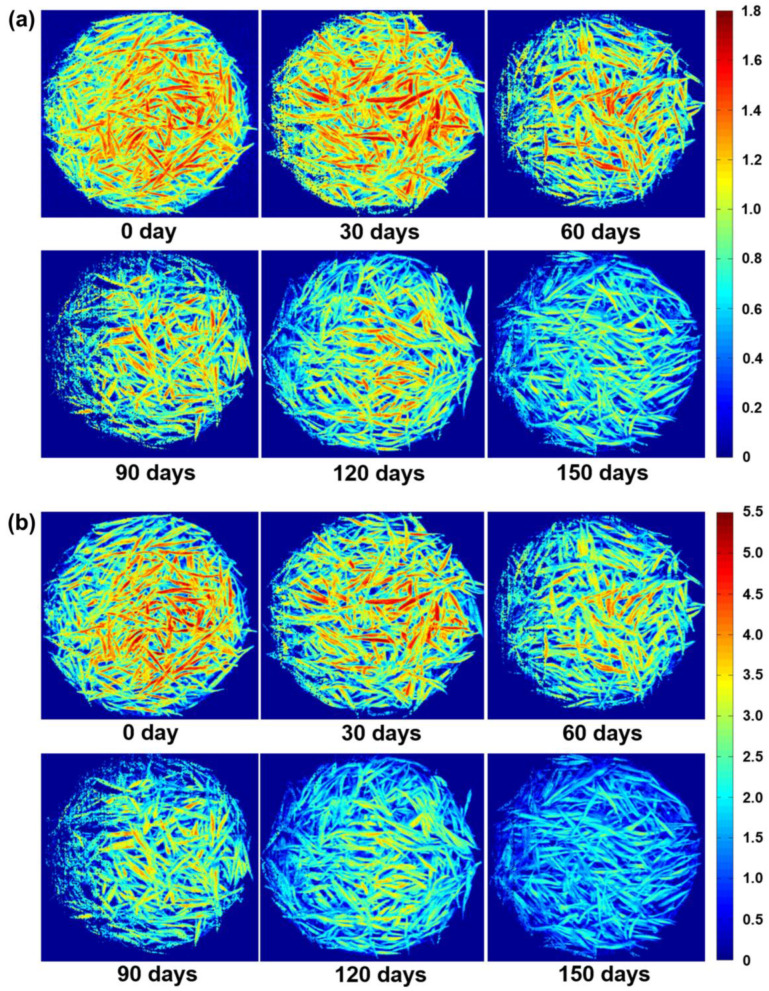
Distribution visualization maps of total saturated (**a**) and total unsaturated fatty acids (**b**) in tea samples with different storage duration.

The optimized CARS-PLS model was used to visualize these two indicators in green tea during storage using total unsaturated fatty acids (Figure 5a) and total saturated fatty acids (Figure 5b). The reflections of every pixel were imported into the constructed CARS-PLS models to automatically generate distribution maps of saturated and unsaturated fatty acids [11]. On this basis, the change in green tea from fresh to not fresh during storage can be clearly observed. Such a visual distribution cannot be obtained by conventional chemical analysis and NIR spectroscopy. Therefore, HSI can be combined with distribution maps for the nondestructive prediction of fatty acid content and spatial distribution in green tea in storage, thus providing a fast and nondestructive option for the rapid evaluation of green tea freshness.

## 4. Conclusions

In this study, chemical imaging was employed for the fast and nondestructive evaluation of green tea freshness during storage. The PLS models failed to predict unsaturated fatty acids and saturated fatty acids with low accuracies. The simplified CARS-PLS yielded satisfactory performance for palmitic acid, total saturated fatty acids, oleic acid, linoleic acid, linolenic acid, and total unsaturated fatty acids, with RPD values of 2.73, 2.26, 3.14, 2.43, 3.01, and 2.78, respectively. Finally, the distribution maps of saturated fatty acids and unsaturated fatty acids were plotted to visualize the changes in fatty acids in green tea samples during storage, thus providing a fast and nondestructive option for the rapid evaluation of green tea freshness.

## Figures and Tables

**Table 1:** Model performance for fatty acid-profile prediction using PLS and CARS-PLS.

Component.	Model	NV	LV	Calibration Set		Prediction Set		
				Rc	RMSEC	Rp	RMSEP	RPD
Palmitic acid	PLS	508	5	0.8561	0.0700	0.7755	0.0848	1.58
	CARS-PLS	22	6	0.9112	0.0556	0.9283	0.0492	2.73
Stearic acid	PLS	508	5	0.6245	0.0276	0.5546	0.0309	1.14
	CARS-PLS	13	4	0.7178	0.0245	0.5914	0.0302	1.17
Arachidic acid	PLS	508	5	0.8120	0.0070	0.4263	0.0010	0.98
	CARS-PLS	6	3	0.8231	0.0005	0.5336	0.0010	1.13
Saturated fatty acids	PLS	508	5	0.8310	0.0910	0.7519	0.1070	1.49
	CARS-PLS	48	7	0.9011	0.0710	0.8961	0.0708	2.26
9-hexadecenoic acid	PLS	508	6	0.8732	0.0007	0.7524	0.0009	1.49
	CARS-PLS	34	12	0.9140	0.0006	0.8653	0.0007	1.98
Oleic acid	PLS	508	6	0.8829	0.0030	0.8493	0.0034	1.91
	CARS-PLS	25	9	0.9230	0.0024	0.9468	0.0021	3.14
Linoleic acid	PLS	508	6	0.8624	0.0839	0.8346	0.0944	1.84
	CARS-PLS	43	8	0.9078	0.0695	0.9094	0.0716	2.43
Linolenic acid	PLS	508	6	0.8643	0.1370	0.8561	0.1390	1.96
	CARS-PLS	43	7	0.9121	0.1110	0.9421	0.0906	3.01
cis-11-Eicosenoicacid	PLS	508	6	0.8189	0.0012	0.7949	0.0013	1.60
	CARS-PLS	13	12	0.8189	0.0012	0.8081	0.0013	1.61
Unsaturated fatty acids	PLS	508	6	0.8653	0.2210	0.8509	0.2310	1.93
	CARS-PLS	43	8	0.9193	0.1720	0.9307	0.1610	2.78

NV—number of variables; LV—number of latent variables; Rc—correlation coefficients of calibration; Rp—correlation coefficients of prediction; RMSEC—root means square error of calibration; RMSEP—root means square error of prediction; RPD—residual prediction deviation. The bold values in the table indicate the optimal prediction model for the fatty acid profile.

## Data Availability

Data are available from the corresponding author upon request.

## Supplementary Materials

The following are available online at https://www.mdpi.com/article/10.3390/foods12020282/s1, Figure S1: Raw spectral curves of all green tea samples in the range of 900–1700 nm.
