# Supplementary figures and images for: Visual Monitoring of Fatty Acid Degradation during Green Tea Storage by Hyperspectral Imaging
Source: Foods. 2023 Jan 7;12(2):282. doi: 10.3390/foods12020282 (PMC9857679; doi:10.3390/foods12020282)

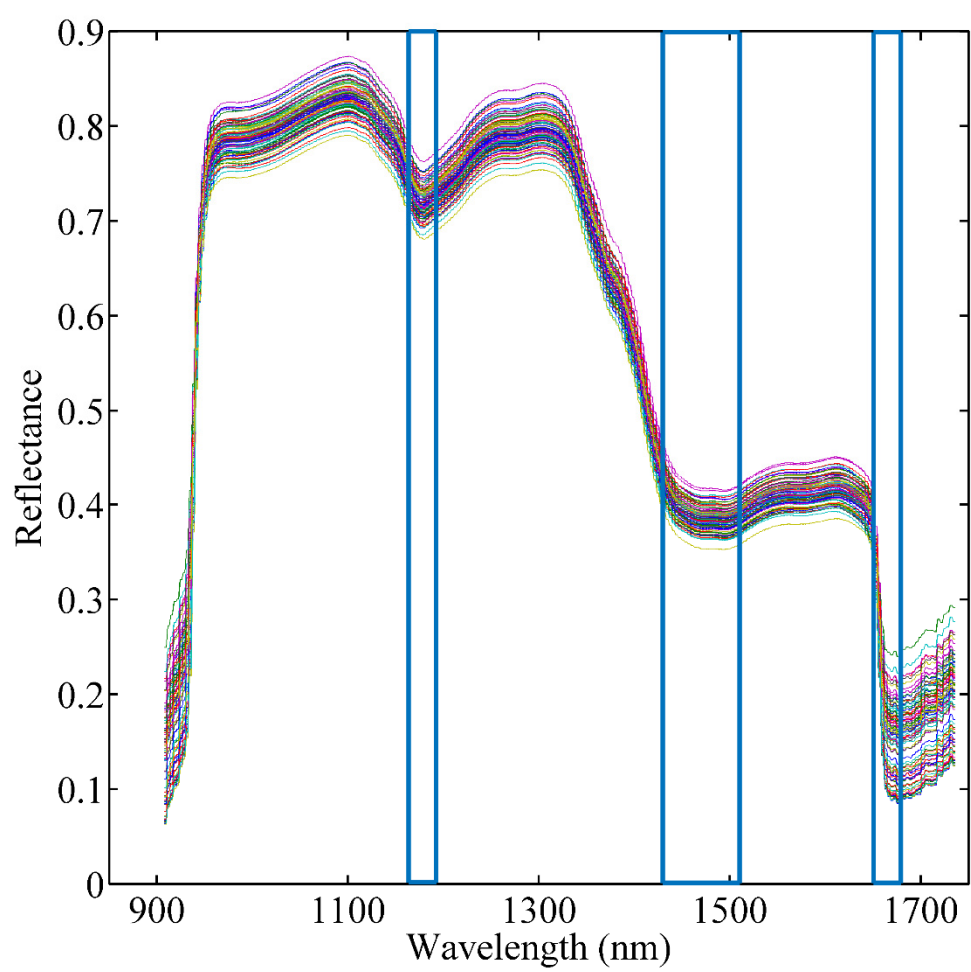

**Figure. S1.** Raw spectral curves of all green tea samples in the range of 900-1700 nm.

Supplement: Supplementary file 1 [file foods-12-00282-s001.zip › foods-2072253-supplementary.pdf]
